# Features of childhood cancer in primary care: a population-based nested case–control study

**DOI:** 10.1038/bjc.2011.600

**Published:** 2012-01-12

**Authors:** R M Dommett, M T Redaniel, M C G Stevens, W Hamilton, R M Martin

**Affiliations:** 1 School of Clinical Sciences, University of Bristol, Level 6 UHB Education Centre, Upper Maudlin Street, Bristol BS2 8AE, UK; 2 Department of Paediatric Oncology, Bristol Royal Hospital for Children, Upper Maudlin Street, Bristol BS2 8BJ, UK; 3 School of Social and Community Medicine, University of Bristol, Canynge Hall, 39 Whatley Road, Bristol BS8 2PS, UK; 4 Peninsula College of Medicine and Dentistry, Universities of Exeter and Plymouth, Veysey Building, Exeter EX2 4SG, UK

**Keywords:** childhood cancer, diagnosis, primary healthcare

## Abstract

**Background:**

This study investigated the risk of cancer in children with alert symptoms identified in current UK guidance, or with increased consultation frequency in primary care.

**Methods:**

A population-based, nested case–control study used data from the General Practice Research Database. In all, 1267 children age 0–14 years diagnosed with childhood cancer were matched to 15 318 controls. Likelihood ratios and positive predictive values (PPVs) were calculated to assess risk.

**Results:**

Alert symptoms recorded in the 12 and 3 months before diagnosis were present in 33.7% and 27.0% of cases *vs* 5.4% and 1.4% of controls, respectively. The PPV of having cancer for any alert symptom in the 3 months before diagnosis was 0.55 per 1000 children. Cases consulted more frequently particularly in the 3 months before diagnosis (86% cases *vs* 41% controls). Of these, 36% of cases and 9% of controls had consulted 4 times or more. The PPV for cancer in a child consulting 4 times or more in 3 months was 0.13 per 1000 children.

**Conclusion:**

Alert symptoms and frequent consultations are associated with childhood cancer. However, individual symptoms and consultation patterns have very low PPVs for cancer in primary care (e.g., of 10 000 children with a recorded alert symptom, approximately 6 would be diagnosed with cancer within 3 months).

Cancer in children is rare, with an annual incidence among 0- to 14-year olds in the United Kingdom of just under 1.4 per 10 000 (Cancer Research UK, 2010) and provides a major diagnostic challenge to all clinicians, particularly those in primary care. Delayed diagnosis may worsen survival; at the very least it reduces the confidence of patients and parents in their doctor (Dixon-Woods *et al*, 2001; Craft and Pritchard-Jones, 2007; Larsen *et al*, 2011). Improving early diagnosis is a priority in the UK Cancer Reform Strategy (Department of Health, 2007, 2011) and other parts of Europe (Olesen *et al*, 2009). As most patients see their GP before diagnosis, the focus of research should be in primary care.

The UK National Institute for Health and Clinical Excellence (NICE) is an independent organisation responsible for providing national guidance on promoting good health and preventing and treating ill health. They have produced guidelines, which describe symptoms that should alert GPs to consider cancer seriously (National Institute for Health and Clinical Excellence, 2005), including cancer in children. These were first published in 2000, and updated in 2005. Lists of alert symptoms are provided for each childhood cancer and some are stratified by age; for example, unexplained hepatosplenomegaly at any age is listed as indicating immediate referral for investigation of leukaemia or lymphoma. However, they were developed in the absence of any supporting primary care research in children, and thus largely describe symptoms deemed pathognomic of cancer at the time of diagnosis in tertiary care. How relevant such symptoms are to children in primary care (and earlier in their diagnostic pathway) is unknown. The aim of this study was to investigate the risk of cancer in children with alert symptoms and increased consultation frequency as described in current NICE guidance.

## Materials and Methods

### Study design

We undertook a population-based case–control study nested within a cohort of children registered with the UK General Practice Research Database (GPRD) (www.gprd.com). The GPRD is a prospectively gathered, anonymised database that holds longitudinal administrative, clinical and prescribing records (including all consultations and diagnoses) of 11 million patients, from over 600 general practices across the United Kingdom (covering approximately 8% of the population) (General Practice Research Database, 2011). Individuals registered on the database are representative of the UK population in terms of age, sex and geographical distribution (Office for National Statistics, 2000a). Data are subject to thorough validation (Herrett *et al*, 2010; Khan *et al*, 2010), audit and quality checks, and have been used in >800 peer reviewed publications, including studies to identify and quantify the symptoms of colorectal (Lawrenson *et al*, 2006; Hamilton *et al*, 2008, 2009) and brain tumours (Hamilton and Kernick, 2007), and alarm symptoms in adult cancers (Jones *et al*, 2007).

### Study population

The sample comprised all children aged 0–14 years, inclusive, drawn from all general practices contributing research-standard data to the GPRD between 1 January 1988 and 31 December 2010. To be included, the practices had to have been contributing research-standard data for a minimum of 1 year before each child's date of cancer diagnosis or the index date (see below) for matched controls.

### Cases and controls

The cases were diagnosed with the following cancers: leukaemia, lymphoma, neuroblastoma, soft tissue sarcoma, hepatic, renal, bone and central nervous system tumours, using pre-defined medical codes used in the GPRD (available from investigators). The date of diagnosis for cases was defined as the date of pathological diagnosis, but if this was unavailable, we used the date of the first cancer code entered in the GPRD. Up to 13 controls (children with no diagnosis of cancer at any time) were selected per case, using a computer-generated random sequence, matched on age (within 1 year), sex and practice, and had to be currently registered on the date of diagnosis of their matched case (the index date). Matching was performed on age and sex, as these are strongly associated with consultation rates, and practice, because each practice has its own protocols and/or conventions for record keeping.

The GPRD restricts its data sets to 100 000 individuals for projects funded through the Medical Research Council licence agreement. This restriction mandated a case–control rather than cohort design to ensure we identified sufficient cases of cancer for each particular symptom to provide sufficient power to detect associations (see power calculations below).

### Symptoms and consultations

The GPRD uses just over 100 000 medical codes to encompass all primary care events, including both symptoms and diagnoses. From this list, we assembled libraries of codes representing individual alert symptoms derived from the NICE referral guidelines for suspected cancer in children: these were compiled separately by RMD and WH, and differences agreed by consensus. Two conditions that we considered to be unrelated to cancer were included (head lice, acne) to identify and quantify any recording bias (whereby patients with cancer attend more frequently, so have more opportunities for a symptom to be recorded). In the absence of recording bias, we would anticipate no association of the recording of head lice or acne with subsequent diagnosis of cancer. In contrast, in the presence of better recording among cancer cases, we might have seen a general increase in recording for all symptoms, irrespective of whether they could be plausibly related to the cancer.

Consultations in the 12 months before diagnosis were identified, *a priori*, over three time periods before the index date: 0–3 months, 4–6 months and 7–12 months.

### Analysis

Participants were stratified by age-group (0–4 years and 5–14 years). The magnitude of associations of alert symptoms and patterns of consultation frequency with cancer were identified using univariable conditional logistic regression. To assess the value of symptoms and consultation patterns in diagnosing cancer, likelihood ratios (LRs) and positive predictive values were calculated (Hamilton, 2010). We calculated the PPV using Bayes’ theorem, whereby posterior odds=prior odds × LR (Knottnerus, 2002). We estimated the prior odds from national incidence figures for 2008 (ISD Online, 2009; Northern Ireland Cancer Registry, 2009; Office for National Statistics, 2009; Welsh Cancer Intelligence and Surveillance Unit, 2009), expressed as the odds of developing cancer in 1 year. Annual incidence figures were divided by 4 for the analyses of 3-month time periods. All analyses were performed using Stata, version 10 (Stata Corporation, College Station, TX, USA, 2008).

### Power calculation

Sample sizes were predetermined by the cancer numbers in the GPRD, so we performed a power calculation, with two-sided 5% significance. An estimated 350 cases (e.g., of leukaemia) and 13 controls per case provided over 99% power to identify a change in the prevalence of a rare variable from 5% in controls to 10% in cases. For rarer cancers, an estimated 80 cases (e.g., of neuroblastoma) and 13 controls per case provided over 84% power to identify a change in a rare variable from 5% in controls to 15% in cases and 97% power to identify a change in a common variable from 30 to 50%.

## Results

In all, 1267 eligible cases of childhood cancer and a corresponding 15 318 eligible controls were identified. Their diagnoses, age-groups and gender are summarised in Table 1. In line with UK demography (Office for National Statistics, 2000b), 34% of cases were aged 0–4 compared with 31% of controls; 55% of cases and controls were male; and 83% of cases and controls were from England.

Table 2 shows the overall frequency of recorded alert symptoms for the total study, along with odds ratios (ORs), LRs and PPVs. In all, 27.0% of cases had a recorded alert symptom in the 3 months before diagnosis compared with 1.4% of controls (OR: 28.8; 95% CI: 23.5, 35.3). In the year before diagnosis, 33.7% of cases had any alert symptom recorded compared with 5.4% of controls (OR: 9.8; 95% CI: 8.5, 11.4). Thus, having an alert symptom is associated with an increased odds of cancer of up to 28.8-fold compared with consulting without an alert symptom. The LRs were 19.6 and 6.2 for symptoms in the 3 months or 12 months before diagnosis, respectively, suggesting that alert symptoms should alter the prior probabilities somewhat. However, the PPV of having cancer in a patient consulting with any alert symptom in the 0- to 3-month period was 0.55 per 1000 children and in the 12-month period was 0.70 per 1000 children. Thus, of 10 000 children with a recorded alert symptom, only up to 6 would be diagnosed with cancer within 3 months. This low PPV is because the prior probability of cancer in this age-group is very small (approximately 1.4 in 10 000 per annum and 0.35 in 10 000 in a 3-month period (Cancer Research UK, 2010). Results were similar when stratified by age-group.

Table 3 shows the frequencies, magnitudes of associations (ORs) and diagnostic performance (LRs and PPVs) of specific symptoms. Neurological symptoms, excluding headache, were most frequently recorded in all cases, followed by headache and lymphadenopathy. Headache was more common in the older age-group and lymphadenopathy in the younger age-group. Although the LRs range from 4.9 to 169.3, the PPVs are low. Hepatosplenomegaly had the highest PPV: of 10 000 children with a record of hepatosplenomegaly, our estimates suggest that 219 would have cancer. The OR and LR for the control conditions (acne and head lice) were 1.0.

Table 4 shows consultation rates. Cases consulted more than controls in the year before diagnosis. This was consistent across all diagnostic groups. Differences in consultation rates between cases and controls were most apparent in the 3 months immediately before diagnosis with cases having a median of three consultations (interquartile range (IQR) 2–4) compared with one consultation (IQR 1–2) in controls.

Among cases, 86.0% had seen their GP in the 3 months before cancer diagnosis compared with 40.9% of controls (OR: 10.1; 95% CI: 8.5, 11.9). Of these, 35.5% of cases had consulted 4 times or more compared with 9.1% of the controls (OR: 12.4; 95% CI: 10.0, 15.3). Thus, consulting 4 times or more in 3 months is associated with an increased odds of cancer of up to 12.4-fold compared with consulting just once. However, the PPV for childhood cancer in a patient consulting 4 times or more in 3 months was only 0.01%. Thus, of 10 000 children consulting 4 times or more in 3 months, only 1 would be diagnosed with cancer (compared with a prior probability of cancer of 0.35 in 10 000 in a 3-month period) (Cancer Research UK, 2010).

## Discussion

This study confirms an association between alert symptoms and childhood cancer. Every symptom in NICE guidance was more commonly recorded in cases than controls. However, individual symptoms were relatively uncommonly recorded in cases: overall just over a quarter of cases had any alert symptom recorded in the 3 months, and only a third had one in the year, before diagnosis. Alert symptoms were also recorded in controls: this, coupled with the rarity of childhood cancer, meant that any individual symptom had a very low PPV for cancer in primary care. Only hepatosplenomegaly had a PPV above 10 per 1000 children, but even this was based on only 14 cases. Children with cancer see their doctors more frequently, particularly in the 3 months before diagnosis. Even so, the absolute risk of cancer in a patient consulting four or more times is only 0.13 per 1000.

### Strengths and weaknesses

This is the first study of childhood cancer to use primary care data that has been collected prospectively. It is large, and practices in the GPRD are broadly representative of the UK population (General Practice Research Database, 2011), so our results should be generalisable. The breakdown of cancers generally matched nationally reported figures, with leukaemia the most common diagnosis overall and CNS tumours the most common solid tumour. Our cohort had a larger than expected number of bone tumours, which was most apparent, as expected, in the 5- to 14-year age-group (Stiller *et al*, 2007). In comparison with national age-specific statistics for 2010 (Cancer Research UK, 2010), there appeared to be an under-ascertainment of some cancers in the 0–4 age-group, by approximately 10% for leukaemia, lymphoma, brain, soft tissue sarcoma and neuroblastoma. Such under-ascertainment could influence the magnitude of the associations we observed for consultation frequency and alert symptoms with cancer, if these variables were related to the likelihood of cancer being recorded in the GPRD. This could occur, for example, if those children without an alert symptom or fewer consultations were less likely to have their final cancer diagnosis recorded in the GPRD than those with an alert symptom. However, if this were the case, we would have overestimated the PPVs for alert symptoms and hence this potential limitation does not detract from our main finding that individual symptoms and consultation patterns have low PPVs for cancer in primary care.

The data were collected prospectively, precluding recall bias. Recording bias is a theoretical possibility (e.g., when features of cancer are preferentially recorded in patients who turn out to have cancer), but it is more likely that symptoms are under-recorded in the GPRD (doctors preferring to record diagnoses where possible). Under-recording should not affect LRs (which underpin PPVs) as long as it is consistent between cases and controls; we have no reason to think this is not the case. The OR and LR of 1 for the control conditions (head lice or acne) suggests no recording bias in relation to patients with cancer attending more frequently, so having more opportunities for a symptom to be recorded.

We derived alert symptoms from the NICE referral guidance for childhood cancer (National Institute for Health and Clinical Excellence, 2005). However, because of the limitations of electronic coding it is difficult to capture the nuances of both the clinical consultation and the NICE guidance regarding symptom intensity and progression. Instinct has a part in all diagnosis, with cancer being no exception (Stolper *et al*, 2009) (especially rare cancers, as GPs do not encounter enough cases to become experienced): this will not have been captured by this study.

### Comparison with previous literature

The low incidence of alert symptoms in the control population is consistent with findings in teenagers and young adults suggesting such symptoms are reported in only 4% of consultations (Fern *et al*, 2011). Increased primary care consultation rates have been reported in children diagnosed with brain tumours up to 6 months before diagnosis (Ansell *et al*, 2010). Our data are also consistent with the observation that parents will continue to return to their GP because their instinct is that their child ‘is not right’ although they may be unable to identify a specific problem (Dixon-Woods *et al*, 2001).

### Implication of the findings

Having any alert symptom does alter the prior probability of cancer in the subsequent 3 months, from the underlying rate of around 0.35 in 10 000 in 3 months among 0- to 14-year olds (Cancer Research UK, 2010) to 6 in 10 000. Our data suggest, however, that the current NICE guidelines have a limited role in primary care, as their predictive value for childhood cancer is so low. The problem is not with the symptom list or the fact that it was derived from tertiary care data – as all symptoms were associated with cancer – but with how a GP is supposed to select children for investigation by a paediatrician. It is unrealistic to suggest that all patients with alert symptoms should be referred. Instead, GPs will have to use additional clues, such as an increased consultation frequency (with four consultations in 3 months appearing a reasonable starting point), abnormal examination findings, multiple symptoms and their instinct. We could not study parental concern, although this is potentially relevant too. One advantage of primary care is the ease of review: ‘safety-netting’ is increasingly recognised as important in adult cancer diagnosis – and may be even more pertinent with children (Almond *et al*, 2009). This may be particularly relevant if patients consult different GPs with what appear to be independent illnesses or complaints. Our findings support the NICE recommendation of urgent referral for a child presenting several times with the same problem, but no clear diagnosis (National Institute for Health and Clinical Excellence, 2005).

### Conclusion

Alert symptoms and frequent consultations are associated with childhood cancer. However, individual symptoms and consultation patterns have very low PPVs for cancer in primary care.

Future studies, including qualitative ones, may help to define the symptoms of cancer in children further. Although these would necessarily be retrospective, they could include the parents and clinicians. This may allow better precision of what symptoms actually matter. Until then, we have a fairly blunt instrument of NICE guidance to help with selection of children for investigation. What is clear is that the current position is unsatisfactory: delays (or perceived delays) in diagnosis can have major implications on acceptance of a cancer diagnosis and a patient and family's subsequent healthcare-seeking behaviour.

## Figures and Tables

**Table 1:** Distribution of cases and controls by cancer site, age and gender

	**All patients**				**By age-groups**								**By gender**							
					**0–4**				**5–14**				**Male**				**Female**			
	**Case (*N*=1267)**		**Control (*N*=15 318)**		**Case (*N*=436)**		**Control (*N*=4802)**		**Case (*N*=831)**		**Control (*N*=10 516)**		**Case (*N*=703)**		**Control (*N*=8461)**		**Case (*N*=564)**		**Control (*N*=6857)**	
**Cancer site**	**Freq.**	**Percent**	**Freq.**	**Percent**	**Freq.**	**Percent**	**Freq.**	**Percent**	**Freq.**	**Percent**	**Freq.**	**Percent**	**Freq.**	**Percent**	**Freq.**	**Percent**	**Freq.**	**Percent**	**Freq.**	**Percent**
Leukaemia	368	29.0	4484	29.3	152	34.9	1763	36.7	216	26.0	2721	25.9	203	28.9	2470	29.2	165	29.3	2014	29.4
Brain	270	21.3	3304	21.6	73	16.7	817	17.0	197	23.7	2487	23.6	141	20.1	1703	20.1	129	22.9	1601	23.3
Lymphoma	142	11.2	1780	11.6	14	3.2	157	3.3	128	15.4	1623	15.4	92	13.1	1152	13.6	50	8.9	628	9.2
Bone	107	8.4	1360	8.9	6	1.4	77	1.6	101	12.2	1283	12.2	62	8.8	787	9.3	45	8.0	573	8.4
Soft tissue sarcoma	91	7.2	1092	7.1	29	6.7	297	6.2	62	7.5	795	7.6	56	8.0	662	7.8	35	6.2	430	6.3
Renal	82	6.5	947	6.2	59	13.5	655	13.6	23	2.8	292	2.8	40	5.7	443	5.2	42	7.4	504	7.4
Neuroblastoma	75	5.9	828	5.4	52	11.9	538	11.2	23	2.8	290	2.8	45	6.4	494	5.8	30	5.3	334	4.9
Other ICD codes	132	10.4	1523	9.9	51	11.7	498	10.4	81	9.7	1025	9.7	64	9.1	750	8.9	68	12.1	773	11.3
Total					436	100.00	4802	100.00	831	100.00	10 516	100.00	703	100.00	8461	100.00	564	100.00	6857	100.00

Abbreviation: ICD=international classification of disease. The rows show the number of controls matched to the specific cancer cases, for example, 368 children with leukaemia were matched to 4484 non-cancer controls, while 270 children with brain cancer were matched to 3304 non-cancer controls, and so on.

**Table 2:** The association of having any NICE alert symptom and a diagnosis of cancer, all cases

	**Cases (*N*=1267)**		**Control (*N*=15 318)**						
**Age-group, period before diagnosis**	**Freq.**	**%**	**Freq.**	**%**	**OR (95% CI)**	**Sensitivity**	**Specificity**	**Likelihood ratio**	**Positive predictive value (per 1000) (95% CI)**
*All ages*									
0–3 Months	342	26.99	211	1.38	28.8 (23.5–35.3)	27.0	98.6	19.60	0.55 (0.47–0.65)
0–12 Months	427	33.70	829	5.41	9.8 (8.5–11.4)	33.7	94.6	6.23	0.70 (0.64–0.78)
									
*0–4 Year age-group*									
*N*	436		4802						
0–3 Months	96	22.02	55	1.15	25.2 (17.3–36.7)	22.0	98.9	19.22	0.81 (0.59–1.12)
0–12 Months	124	28.44	248	5.16	8.1 (6.2–10.5)	28.4	94.8	5.51	0.93 (0.77–1.13)
									
*5–14 Year age-group*									
*N*	831		10 516						
0–3 Months	246	29.60	156	1.48	30.4 (23.8–38.7)	29.6	98.5	19.96	0.56 (0.47–0.68)
0–12 Months	303	36.46	581	5.52	10.7 (9.0–12.8)	36.5	94.5	6.60	0.75 (0.66–0.84)

Abbreviations: CI=confidence interval; NICE= National Institute for Health and Clinical Excellence; OR=odds ratio. All case–control comparisons were strongly significant with *P*-values <0.001.

**Table 3:** The association of selected alert symptoms and a diagnosis of cancer, all cases, 0–12 months

	**Cases**		**Control**				
**Symptom**	**Freq.**	**%**	**Freq.**	**%**	**OR (95% CI)^a^**	**Likelihood ratio**	**Positive predictive value (per 1000) (95% CI)**
*All cases*							
*N*	1267		15 318				
Alert symptoms							
Neurological symptoms	108	8.52	207	1.35	7.0 (5.5–8.9)	6.31	0.83 (0.67–1.05)
Headache	90	7.10	224	1.46	5.6 (4.3–7.3)	4.86	0.64 (0.51–0.82)
Lymphadenopathy	82	6.47	136	0.89	8.2 (6.2–11.0)	7.29	0.96 (0.74–1.26)
Lump/mass/swelling	56	4.42	52	0.34	14.2 (9.5–21.1)	13.02	1.72 (1.19–2.50)
Fatigue	47	3.71	88	0.57	6.8 (4.7–9.8)	6.46	0.85 (0.60–1.21)
Back pain	40	3.16	73	0.48	7.6 (5.1–11.3)	6.62	0.88 (0.60–1.28)
Bruising	38	3.00	76	0.50	6.0 (4.1–9.0)	6.04	0.80 (0.54–1.18)
Urinary symptoms	15	1.18	9	0.06	21.0 (9.2–47.9)	20.15	2.66 (1.17–6.09)
Hepatosplenomegaly	14	1.10	1	0.01	149.6 (19.6–1142.6)	169.26	21.9 (2.95–170.34)
Control conditions (acne, head lice)	23	1.82	278	1.81	1.0 (0.7–1.6)	1.00	0.13 (0.09–0.20)
							
*0–4 Year age-group*							
*N*	436		4802				
Alert symptoms							
Neurological symptoms	43	9.86	105	2.19	5.3 (3.6–7.7)	4.51	0.76 (0.54–1.07)
Headache	8	1.83	11	0.23	8.9 (3.6–22.2)	8.01	1.35 (0.55–3.35)
Lymphadenopathy	20	4.59	61	1.27	4.1 (2.4–7.0)	3.61	0.61 (0.37–1.00)
Lump/mass/swelling	16	3.67	15	0.31	11.6 (5.6–24.1)	11.75	1.98 (0.99–3.99)
Fatigue	15	3.44	32	0.67	5.8 (3.0–11.0)	5.16	0.87 (0.48–1.60)
Back pain	4	0.92	4	0.08	12.9 (3.2–51.5)	11.01	1.86 (0.47–7.42)
Bruising	20	4.59	24	0.50	9.3 (5.0–17.3)	9.18	1.55 (0.86–2.79)
Urinary symptoms	8	1.83	2	0.04	49.2 (10.4–231.6)	44.06	7.39 (1.59–34.96)
Hepatosplenomegaly	7	1.61	1	0.02	67.1 (8.1–554.4)	77.10	12.86 (1.61–105.69)
Control conditions (acne, head lice)	8	1.83	50	1.04	2.0 (0.9–4.3)	1.76	0.30 (0.14–0.62)
							
*5–14 Year age-group*							
*N*	831		10 516				
Alert symptoms							
Neurological symptoms	65	7.82	102	0.97	8.6 (6.3–11.9)	8.06	0.91 (0.67–1.23)
Headache	82	9.87	213	2.03	5.4 (4.1–7.1)	4.87	0.55 (0.43–0.70)
Lymphadenopathy	62	7.46	75	0.71	11.7 (8.2–16.6)	10.46	1.18 (0.85–1.64)
Lump/mass/swelling	40	4.81	37	0.35	15.4 (9.6–24.7)	13.68	1.54 (0.99–2.40)
Fatigue	32	3.85	56	0.53	7.4 (4.8–11.5)	7.23	0.82 (0.53–1.25)
Back pain	36	4.33	69	0.66	7.2 (4.7–11.0)	6.60	0.75 (0.50–1.11)
Bruising	18	2.17	52	0.49	4.5 (2.6–7.6)	4.38	0.49 (0.29–0.84)
Urinary symptoms	7	0.84	7	0.07	12.8 (4.5–36.4)	12.65	1.43 (0.50–4.07)
Hepatosplenomegaly	7	0.84	0	0.00			
Control (acne, head lice)	15	1.81	228	2.17	0.8 (0.5–1.4)	0.83	0.09 (0.06–0.16)

Abbreviations: CI=confidence interval; OR=odds ratio.

a Compared with those without symptoms; computed using conditional logistic regression.

All alert symptom case–control comparisons were strongly significant with *P*-values <0.001.

Neurological symptoms – seizures, reduced conscious level, cranial nerve abnormalities, visual disturbances, gait abnormalities, motor or sensory signs, unexplained deteriorating school performance or developmental milestones, unexplained behavioural and/or mood disturbance.

Urinary symptoms – retention, haematuria.

**Table 4:** The association between the number of consultations^a^ and a diagnosis of cancer, 0–3 months before diagnosis

	**Case**		**Control**				
**Number of consultations**	**Freq.**	**%^b^**	**Freq.**	**%^b^**	**OR^c^ (95% CI)**	**Likelihood ratio**	**Positive predictive value (per 1000) (95% CI)**
*All cases*							
*N*	1267		15 318				
No consultations	178	14.0	9056	59.1	1.0		
With consultations	1089	86.0	6262	40.9	10.1 (8.5–11.9)	2.1	0.07 (0.07–0.07)
1	262	24.1	3565	56.9	1.0		
2	252	23.1	1457	23.3	2.7 (2.2–3.3)	1.0	0.03 (0.03–0.04)
3	188	17.3	668	10.7	4.9 (3.9–6.1)	1.6	0.05 (0.05–0.06)
4 or more	387	35.5	572	9.1	12.4 (10.0–15.3)	3.9	0.13 (0.12–0.14)
							
*0–4 Year age-group*							
*N*	436		4802				
No consultations	50	11.5	2093	43.6	1.0		
With consultations	386	88.5	2709	56.4	6.1 (4.5–8.4)	1.6	0.07 (0.06–0.07)
1	82	21.2	1370	50.6	1.0		
2	83	21.5	669	24.7	2.4 (1.7–3.3)	0.9	0.04 (0.03–0.05)
3	68	17.6	335	12.4	4.2 (3.0–6.1)	1.4	0.06 (0.05–0.08)
4 or more	153	39.6	335	12.4	9.8 (7.0–13.7)	3.2	0.14 (0.12–0.16)
							
*5–14 Year age-group*							
*N*	831		10 516				
No consultations	128	15.4	6963	66.2	1.0		
With consultations	703	84.6	3553	33.8	11.9 (9.8–14.5)	2.5	0.07 (0.07–0.07)
1	180	25.6	2195	61.8	1.0		
2	169	24.0	788	22.2	2.8 (2.2–3.6)	1.1	0.03 (0.03–0.04)
3	120	17.1	333	9.4	5.2 (3.9–7.0)	1.8	0.05 (0.04–0.06)
4 or more	234	33.3	237	6.7	14.3 (10.9–18.9)	5.0	0.14 (0.12–0.17)

Abbreviations: CI=confidence interval; GP=general practice; OR=odds ratio.

a All primary care consultations including out of hours and telephone consultations.

b For categories 1, 2, 3 and 4 or more, proportions reflect only patients with consultations.

c OR: represents the odds of being diagnosed with cancer given more consultations with the GP; computed using conditional logistic regression.
